# MAPK-dependent regulation of IL-1- and β-adrenoreceptor-induced inflammatory cytokine production from mast cells: Implications for the stress response

**DOI:** 10.1186/1471-2172-5-22

**Published:** 2004-09-21

**Authors:** David S Chi, S Matthew Fitzgerald, Shannon Pitts, Karen Cantor, Ellis King, Steven A Lee, Shau-Ku Huang, Guha Krishnaswamy

**Affiliations:** 1 Department of Internal Medicine, James H. Quillen College of Medicine, East Tennessee State University, Johnson City, Tennessee 37614, USA; 2 The Johns Hopkins Asthma and Allergy Center, Baltimore, MD 21224, USA

## Abstract

**Background:**

Catecholamines, such as epinephrine, are elaborated in stress responses, and mediate vasoconstriction to cause elevation in systemic vascular resistance and blood pressure. Our previous study has shown that IL-1 can induce mast cells to produce proinflammatory cytokines which are involved in atherogenesis. The aim of this study was to determine the effects of epinephrine on IL-1-induced proatherogenic cytokine production from mast cells.

**Results:**

Two ml of HMC-1 (0.75 × 10^6 ^cells/ml) were cultured with epinephrine (1 × 10^-5 ^M) in the presence or absence of IL-1β (10 ng/ml) for 24 hrs. HMC-1 cultured alone produced none to trace amounts of IL-6, IL-8, and IL-13. IL-1β significantly induced production of these cytokines in HMC-1, while epinephrine alone did not. However, IL-6, IL-8, and IL-13 production induced by IL-1β were significantly enhanced by addition of epinephrine. The enhancing effect appears to involve NF-κB and p38 MAPK pathways. Flow cytometry showed the presence of β_1 _and β_2 _adrenoreceptors on resting mast cells. The enhancing effect of proatherogenic cytokine production by epinephrine was down regulated by the β_1 _and β_2 _adrenoceptor antagonist, propranolol, but not by the β_1 _adrenoceptor antagonist, atenolol, suggesting the effect involved β_2 _adrenoceptors. The enhancing effect of epinephrine on proatherogenic cytokine production was also down regulated by the immunosuppressive drug, dexamethasone.

**Conclusions:**

These results not only confirm that an acute phase cytokine, IL-1β, regulates mast cell function, but also show that epinephrine up regulates the IL-1β induction of proatherogenic cytokines in mast cells. These data provide a novel role for epinephrine, a stress hormone, in inflammation and atherogenesis.

## Background

Atherogenesis involves the cellular infiltration of several cell types, including monocytes, T lymphocytes, and mast cells. Cytokine secretion by these cells and endothelial cells are contributing factors in the growth and propagation of atherosclerotic plaques as well as the stability and degradation of fibrous caps. Cytokines implicated in atherogenesis include Interleukin (IL)-1β, IL-6, IL-8, IL-13, and Tumor Necrosis Factor (TNF) [1,2].

IL-1β is secreted mainly by macrophages and virtually by every cell type in the body. IL-1β is produced in response to various stimulants, such as cytokines, bacteria, and viruses, but most interestingly to epinephrine [3]. IL-1β has a broad range of functions which includes activation of neutrophils, endothelial cells, monocytes, T-cells, and mast cells. It may also induce procoagulant changes in endothelial tissue. IL-6 induces an acute phase response consisting of increased fibrinogen synthesis and thrombocytosis with increased vascular permeability. The detection of IL-6 in the blood of patients suffering from unstable angina suggests that nuclear factor-kappa B (NF-κB) activation may be occurring at the vascular level in patients with heart disease [4-7]. IL-8 is in the CXC family of chemokines and functions to recruit neutrophils to the site of inflammation. IL-13 exerts multiple effects on cell differentiation and function of monocytes/macrophages. It can also suppress the cytotoxic function of monocytes/macrophages and the production of proinflammatory cytokines by these cells [8,9].

Mast cells are found preferentially around blood vessels and beneath the epithelium of the skin and mucus membranes [1,10-12]. Traditionally, mast cells are responsible for allergy and asthma pathogenesis. Typically, mast cell activation occurs in response to cross-linkage of the high affinity IgE receptor (FcεRI) by antigen and IgE [12]. Activation may also occur in response to a range of agents, such as pathogens, cytokines, and even oxidized low density lipoprotein (ox-LDL). After activation, key mediators secreted by mast cells include preformed mediators like histamine, proteoglycans, proteases, and several cytokines and growth factors [1]. Mast cells have been observed in both aortic atherosclerotic lesions and in coronary arteries. The large numbers of mast cells found in the adventitia of arteries and in the intima are in proportion to the severity of heart disease [13]. The study of the distribution, activation, and phenotype of mast cells in lesions of 250 specimens of human carotid arteries by Jeziorski, *et al.* further supports the role of mast cells in atherogenesis [14]. They demonstrated significant numbers and focal accumulations of mast cells in association with macrophages and extensive activation/degranulation at all developmental stages of atherosclerotic lesion development. It now appears likely that inflammatory events and mast cells play an important role in atherogenesis as recently reviewed by us [1,2].

Stress is known to influence immune function [15-17]. An immunoregulatory effect of the sympathetic nervous system in stress has been indicated for some time [18]. Catecholamines, such as epinephrine, norepinephrine, and dopamine, are elevated in stress responses, and mediate vasoconstriction and an increase in blood pressure as a result of increased peripheral vascular resistance. In disorders such as sepsis, cardiovascular disease, or cocaine abuse, catecholamines are elaborated in excess. Sustained increases in circulating catecholamines by infusion of epinephrine or norepinephrine have been shown to cause moderate cardiovascular and metabolic effects [19]. Catecholamines induce aggravation of aortic and coronary atherosclerosis in monkeys [20] and play a direct role in atherogenesis and cardiovascular disease [21].

Epinephrine and norepinephrine increase the uptake of low density lipoprotein in atheroscelotic plaques in rabbits and rats [22] as well as enhance proliferation of rat endothelial and smooth muscle cells [23]. It has been reported that norepinephrine increases adherence and chemotaxis of macrophages [24]. Epinephrine also upregulates the surface expression of L-selectin on monocytes *in vitro *[25]. Most recently, we have reported that nitric oxide production from macrophages induced by LPS is enhanced by catecholamines [26]. Both epinephrine and IL-1 are involved in acute phase responses seen in stress and in coronary artery disease. Studies have shown that norepinephrine can induce IL-1β mRNA in mycocardial tissue [27] and that infusion of IL-1β in animal models can induce expression of catecholamines [28,29]. These data suggest that, in some conditions, both IL-1β and catecholamines can be delivered to tissues that can then mediate additive or modulatory effects. Moreover, as reviewed by Gidron Y et al., [30] stress in conjunction with the release of catecholamines and proinflammatory cytokines, can potentiate atherogenesis. Hence, studies of the interactions between catecholamines, monokines and inflammatory cell activation are especially relevant. The aim of the study was to determine whether epinephrine affects IL-1β induced proatherogenic cytokine production in mast cells, a phenomenon previously not described. Our results indicated that epinephrine synergized with IL-1β in the production of proatherogenic cytokines, suggesting a potential role for this interaction in inflammatory and atherogenic states.

## Results

### Epinephrine enhances IL-1β-induced IL-6, IL-8, and IL-13 production in mast cells

**Figure 1:**
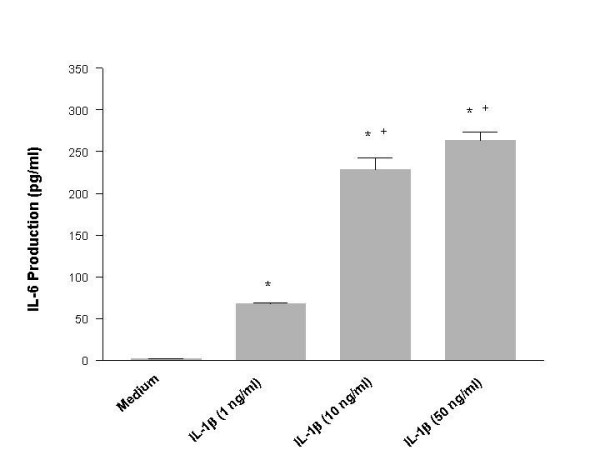
IL-1β induces IL-6 production from HMC-1 cells. To each well of a 6 well culture plate, two ml of HMC-1 mast cells (0.75 × 10^6^cells/ml) were cultured with IL-1β (1, 10, and 50 ng/ml) for 24 hours. The cultures were carried out in triplicate. Supernatants were harvested for measuring IL-6 by ELISA. By Student's t-test analysis, * indicates p < 0.0001, when compared with the medium alone. + indicates p < 0.0005, when compared with the IL-1 (1 ng/ml) group.

**Figure 2:**
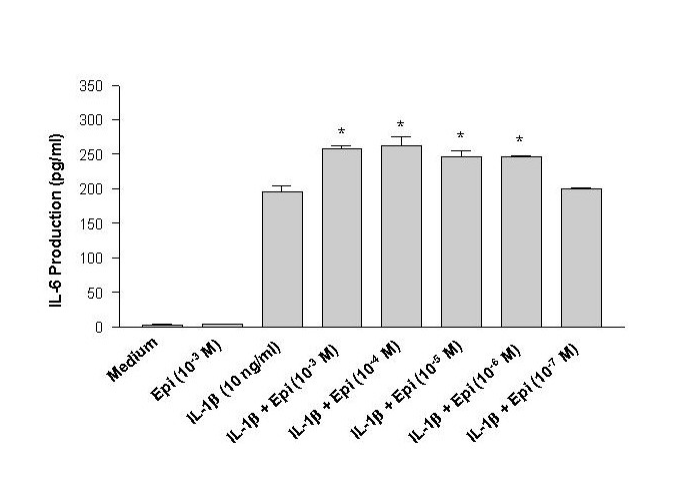
Enhancing effect of epinephrine on IL-6 production from IL-1β-induced HMC-1 cells. To each well of a 6 well culture plate, two ml of HMC-1 mast cells (0.75 × 10^6 ^cells/ml) were cultured with epinephrine (1 × 10^-3 ^to 1 × 10^-7 ^M) in the presence and absence of IL-1β (10 ng/ml) for 24 hrs in triplicate. Supernatants were harvested for measuring IL-6 by ELISA. By Student's t-test analysis, * indicates p < 0.05, when compared with the IL-1β-treated group.

**Figure 3:**
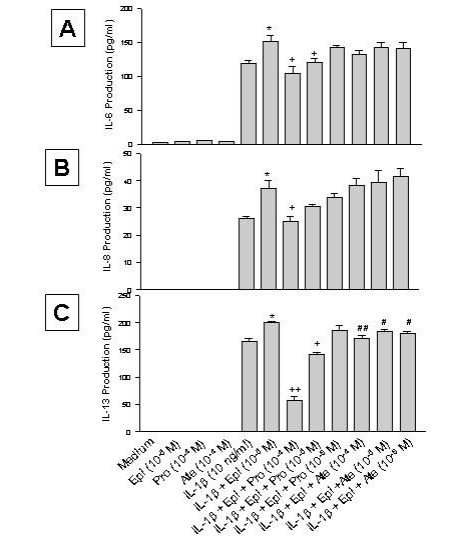
Effect of propranolol (Pro) and atenolol (Ate) on the enhancing effect of epinephrine (Epi) on production of IL-6 (A), IL-8 (B), and IL-13 (C) from IL-1β-induced HMC-1 cells. To each well of a 6 well culture plate, two ml of HMC-1 mast cells (0.75 × 10^6 ^cells/ml) were cultured alone (Medium), or in the presence of IL-1β (10 ng/ml), Epi (1 × 10^-5 ^M), Pro (1 × 10^-4 ^to 1 × 10^-6 ^M), Ate (1 × 10^-4 ^to 1 × 10^-6 ^M), and the combinations of these reagents for 24 hrs in triplicate. Supernatants were harvested for measuring IL-6, IL-8, and IL-13 by ELISA. IL-8 and IL-13 production were not detected in the Medium, Epi, Pro, and Ate alone groups. In A and B, by Student's t-test analysis, * and + indicate p < 0.05, when compared with the IL-1β-treated group, and the IL-1β plus Epi group, respectively. In C, * indicates p < 0.01, when compared with the IL-1β-treated group; p values for ++, +, ##, and # were <0.00005, <0.0005, <0.01, and <0.05, when compared with the IL-1β plus Epi group.

Human mast cell line, HMC-1, was incubated with IL-1β at various concentrations for 24 hours. The cell free supernatants of the cultures were harvested and subjected to IL-6 assay. HMC-1 cultured in medium alone produced trace amounts of IL-6. The IL-6 production from HMC-1 cultures treated with IL-1β was significantly increased in a dose-dependent manner (p < 0.0001) (Fig. 1). Since there was no significant difference in the IL-6 production induced by IL-1β at concentrations of 10 and 50 ng/ml, 10 ng/ml of IL-1β has been used to induce cytokine production in HMC-1 for the rest of the experiments. Epinephrine (Epi) alone at a concentration of 10^-3 ^M did not induce production of IL-6 in HMC-1 (Fig. 2). When epinephrine at 10^-3 ^to 10^-7 ^M concentration was added simultaneously with IL-1β into the cultures, the production of IL-6 was enhanced significantly (p < 0.05) compared with that induced by IL-1β alone (Fig. 2). Since the physiological concentration of epinephrine in plasma is 0.11 – 0.27 × 10^-6 ^M [31], we decided to use epinephrine at a supramaximal concentration of 1 × 10^-5 ^M for the rest of the experiments. In addition to IL-6, the enhancing effect of epinephrine was also observed in the production of IL-8 and IL-13 from IL-1β-induced HMC-1 cells (Fig. 3).

**Figure 4:**
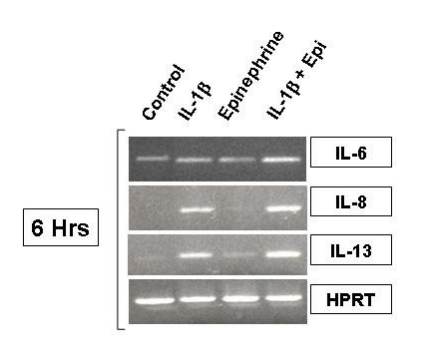
RT-PCR analysis for IL-6, IL-8, and IL-13 in HMC-1 treated with IL-1β and epinephrine. HMC-1 were treated for 6 hours with IL-1β with and without epinephrine and harvested for RNA preparation. RNA was subjected to RT-PCR with specific primers for target genes. HPRT was used as a house keeping gene to ensure equal loading. IL-6 gene expression was increased with IL-1β treatment and further increased with IL-1β plus epinephrine. Epinephrine alone had no effect on IL-6 gene expression in HMC-1. IL-8 and IL-13 showed similar results with a more robust expression of gene transcripts at this time point.

To measure proatherogenic cytokine gene expression, HMC-1 were treated with IL-1β, epinephrine, and IL-1β plus epinephrine for 6 hours and harvested for transcriptional analysis via RT-PCR. IL-1β-treated HMC-1 showed increased IL-6 mRNA transcription as seen with densitometry, while epinephrine alone appeared to have no effect. When IL-1β and epinephrine were added together to HMC-1, IL-6 mRNA expression increased over IL-1β treatment alone (Fig. 4). The intensities of the cytokine and house keeping gene (HPRT) bands were measured by densitometry, and the ratio of the cytokine to the house keeping gene was calculated and assigned as the intensity index. The intensity indices for IL-6 were 0.36 for the control, 0.39 for IL-1β alone, 0.33 for epinephrine alone, and 0.54 for IL-1β plus epinephrine. IL-1β activated IL-8 mRNA production but epinephrine had no effect on IL-8 transcripts. IL-1β and epinephrine treatment together further increased IL-8 mRNA production (Fig. 4). The intensity indices for IL-8 were 0.17 for the control, 0.52 for IL-1β alone, 0.20 for epinephrine alone, and 0.64 for IL-1β plus epinephrine. The results with IL-13 expression showed the same pattern. IL-1β was a good inducer of IL-13 transcription while epinephrine alone only minimally induced IL-13 mRNA. The combined stimulus of IL-1β and epinephrine significantly increased IL-13 mRNA production over that seen with each stimulus alone (Fig 4). Intensity indices for IL-13 were 0.22 for the control, 0.57 for IL-1β alone, 0.20 for epinephrine alone, and 0.64 for IL-1β plus epinephrine. To evaluate further the ability of epinephrine to induce IL-13 transcription at a molecular level, we transiently transfected HMC-1 cells with minimal promoter sequences as described in the materials and methods. IL-1β at 10 ng/ml significantly increased IL-13 promotor activity as detected by luciferase expression (data not shown). Epinephrine did not enhance IL-13 promoter activity suggesting that post-transcriptional mechanisms may be involved in the IL-13 induction. It is likely that epinephrine either prolongs IL-13 mRNA half life and/or enhances IL-13 secretory processes from the mast cell in response to IL-1-stimulation.

### Enhancing effect of epinephrine on proatherogenic cytokine production from IL-1β-induced HMC-1 is down regulated by adrenoceptor antagonists

Since our previous study has shown that the effect of epinephrine on nitric oxide synthesis is mediated by β-adrenoceptors [26], β-adrenergic receptor antagonists (propranolol and atenolol) were used to block the enhancing effect of epinephrine on proatherogenic cytokine production in HMC-1. Propranolol (Pro) and atenolol (Ate) at a concentration of 1 × 10^-4 ^M did not affect the cell viability in the cultures (88 and 90%, respectively, while that of the medium control was 85%), nor induced production of IL-6, IL-8 or IL-13 (Fig. 3). When propranolol at 1 × 10^-4 ^and 1 × 10^-5 ^M was used in the culture, it significantly reduced the enhancing effect of epinephrine on IL-6 production (p < 0.05, Fig. 3A). Propranolol at 1 × 10^-4 ^M also significantly reduced the enhancing effect of epinephrine on IL-8 production (p < 0.05, Fig. 3B), and at 1 × 10^-4 ^and 1 × 10^-5 ^M significantly reduced the enhancing effect of epinephrine on IL-13 production (p < 0.00005 and 0.0005, respectively, Fig. 3C). However, atenolol only significantly reduced the enhancing effect of epinephrine on IL-13 production (p < 0.05, Fig. 3C), but not on IL-6 or IL-8 production (Fig. 3A and 3B).

### Expression of β_1 _and β_2 _adrenergic receptors on mast cells

**Figure 5:**
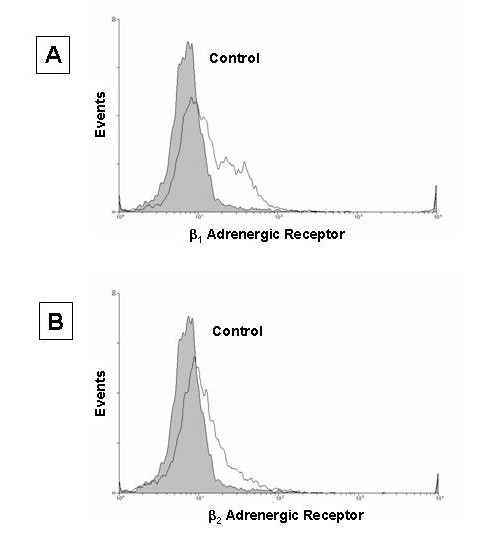
Detection of β_1 _and β_2 _adrenergic receptors on HMC-1 cell by flow cytometry analysis. Resting HMC-1 were harvested and stained with a purified rabbit polyclonal antibody to either β_1 _or β_2 _adrenergic receptor and counter stained with a secondary goat anti-rabbit FITC conjugated antibody. Normal rabbit serum and the FITC conjugated goat anti-rabbit Ig G antibody was used as a staining control.

In order to further identify whether the enhancing effect of epinephrine on proatherogenic cytokine production is through the β-adrenoceptor, HMC-1 cells were incubated with rabbit polyclonal antibodies against β_1 _and β_2 _adrenergic receptors followed by a FITC-labeled second antibody. By flow cytometry analysis, β_1 _and β_2 _adrenergic receptors were found in small amounts on HMC-1 (18.6 and 11.7% respectively) (Fig 5).

### Enhancing effect of epinephrine on proatherogenic cytokine production from IL-1β-induced HMC-1 is down regulated by immunosuppressants

**Figure 6:**
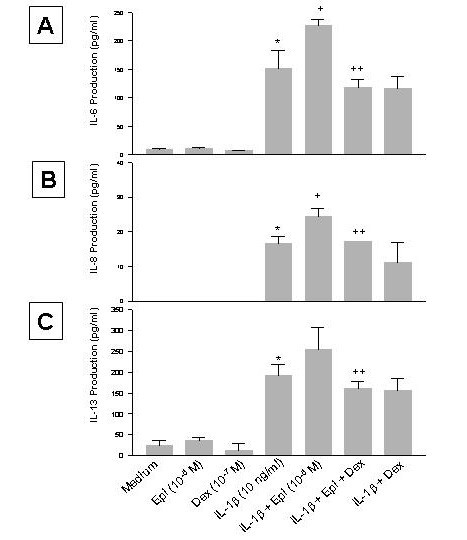
Effect of dexamethasone (Dex) on the enhancing effect of epinephrine (Epi) on production of IL-6 (A), IL-8 (B), and IL-13 (C) from IL-1β-induced HMC-1 cells. To each well of a 6 well culture plate, two ml of HMC-1 mast cells (0.75 × 10^6 ^cells/ml) were cultured alone (Medium), or in the presence of IL-1β (10 ng/ml), Epi (1 × 10^-5 ^M), Dex (1 × 10^-7 ^M), and the combinations of these reagents for 24 hrs in triplicate. Supernatants were harvested for measuring IL-6, IL-8, and IL-13 by ELISA. * p < 0.005, when compared with the medium control, + p < 0.05 compared to the IL-1β-treated group, and ++ p < 0.05 compared to the IL-1β plus Epi group.

Since glucocorticoids are very effective treatment strategies for inflammatory disease, dexamethasone was used to determine its effect on atherogenic cytokine production in HMC-1. Dexamethasone (Dex, 1 × 10^-7 ^M) alone did not induce proatherogenic cytokine production (Fig. 6). However, Dex significantly inhibited the enhancing effect of epinephrine on IL-6 production (p < 0.05, Fig. 6A). The cell viability of the cultures was not different between the medium control (70%) and Dex (64%) groups. Dex also significantly inhibited the enhancing effect of epinephrine on IL-8 and IL-13 production (p < 0.05, Fig. 6B and 6C). When Dex was included in the IL-1β-treatment, it slightly decreased the cytokine production when compared to the IL-1β alone, but the decrease was not significant (Fig. 6).

### Role of NF-κB activation in the enhancing effect of epinephrine on proatherogenic cytokine production from IL-1β-induced HMC-1

**Figure 7:**
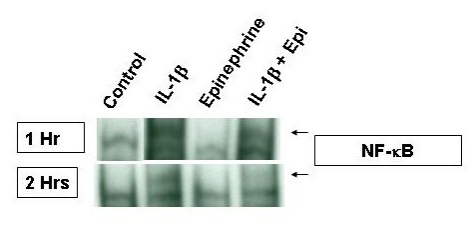
Effects of IL-1β and epinephrine on NF-κB translocation in HMC-1. HMC-1 were treated for 1 and 2 hours with IL-1β and epinephrine. NF-κB translocation was analyzed by a shift in oligonucleotide binding in EMSA gels. After one hour of treatment, NF-κB translocation is increased in the IL-1β treated cells but not in the untreated or epinephrine treated cells. Addition of IL-1β plus epinephrine does not further enhance NF-κB translocation. After two hours of treatment, NF-κB translocation in HMC-1 starts to decrease.

NF-κB is an important transcription factor that mediates the transcription of many proinflammatory cytokine genes. To study the role NF-κB plays in the enhancing effect of epinephrine on proatherogenic cytokine production from IL-1β-induced HMC-1, NF-κB activation was analyzed in HMC-1 cultures. NF-κB translocation, as seen by a shift in oligonucleotide binding in EMSA gels, was not seen in control or epinephrine treated cells (Fig. 7). A marked increase of NF-κB nuclear binding activity was observed in samples stimulated with IL-1β and IL-1β plus epinephrine for one hour but started to diminish after two hours (Fig. 7). Not only did IL-1β plus epinephrine have no further effects on NF-κB translocation over IL-1β treatment alone, it seemed to decrease after one and two hours of stimulation.

### Role of p38 MAPK activation in the enhancing effect of epinephrine on proatherogenic cytokine production from IL-1β-induced HMC-1

**Figure 8:**
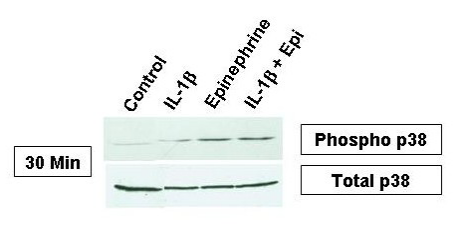
Phosphorylated and total p38 MAPK in HMC-1 cells treated with IL-1β, epinephrine, and IL-1β plus epinephrine. HMC-1 were treated for 30 minutes with the indicated reagents and harvested for phosphorylated p38 expression by Western blot. Unphosphorylated p38 was used as loading control to show total MAPK expression. IL-1β treated cells showed a small amount of p38 activation while the bulk of p38 was activated with epinephrine. IL-1β plus epinephrine had no additional effects over epinephrine alone.

Because of its importance in cytokine signaling, phosphorylated p38 MAPK was also assayed. After 30 minutes of activation, the HMC-1 were lysed to be analyzed for p38 activation by Western blot. The presence of phosphorylated p38 was greatly increased in the epinephrine and IL-1β plus epinephrine samples (Fig. 8). IL-1β alone had small effects on p38 activation at this time point while control levels were virtually nonexistent.

### Enhancing effect of epinephrine on proatherogenic cytokine production from IL-1β-induced HMC-1 is down regulated by NF-κB and p38 MAPK inhibitors

**Figure 9:**
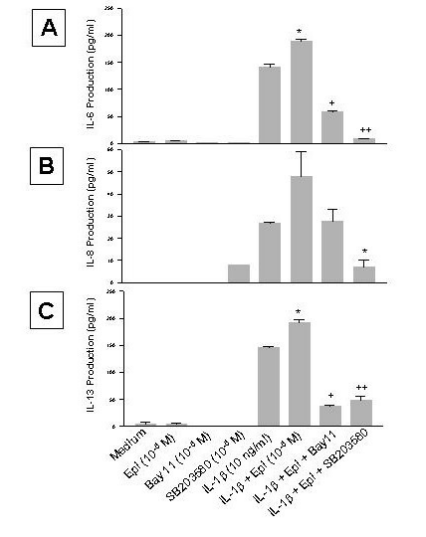
Effect of Bay 11 and SB203580 on the enhancing effect of epinephrine (Epi) on production of IL-6 (A), IL-8 (B), and IL-13 (C) from IL-1β-induced HMC-1 cells. To each well of a 6 well culture plate, two ml of HMC-1 mast cells (0.75 × 10^6 ^cells/ml) were cultured alone (Medium), or in the presence of IL-1β (10 ng/ml), Epi (1 × 10^-5 ^M), Bay 11 (1 × 10^-5 ^M), SB 203580 (1 × 10^-5 ^M), and the combinations of these reagents for 24 hrs in triplicate. Supernatants were harvested for measuring IL-6, IL-8, and IL-13 by ELISA. IL-8 production was not detected in the Medium, Epi, Bay 11 alone groups, while IL-13 production was not detected in the Bay 11 and SB 203580 alone groups. In A, by Student's t-test analysis, * indicates p < 0.005, when compared with the IL-1β-treated group, and + and ++ indicate p < 0.0005 and <0.00005, when compared with the IL-1β plus Epi group. In B, * indicates p < 0.05, when compared with both the IL-1β-treated group, and the IL-1β plus Epi group. In C, * indicates p < 0.005, when compared with the IL-1β-treated group, and + and ++ indicate p < 0.00005 and <0.0001, when compared with the IL-1β plus Epi group.

To confirm the role of NF-κB and p38 MAPK in the enhancing effect of epinephrine on proatherogenic cytokine production from IL-1β-induced HMC-1, Bay 11, an NF-κB inhibitor [32], and SB203580, a specific inhibitor of p38 MAPK [33], were added to the cultures. By themselves, neither Bay 11 (1 × 10^-5 ^M) nor SB203580 (1 × 10^-5 ^M) affected the cell viability of the cultures (92 and 87%, respectively, while that of the medium control was 92%), nor did they induce proatherogenic cytokine production (Fig. 9). However, Bay 11 and SB203580 significantly inhibited the enhancing effect of epinephrine on IL-6 production (p < 0.0005 and p < 0.00005, respectively, Fig. 9A). Bay 11 decreased the IL-1β-epinephrine induced IL-8 production but not significantly, however SB203580 did significantly inhibit the enhancing effect of epinephrine on IL-8 production (p < 0.05, Fig. 9B). Bay 11 and SB203580 significantly inhibited the enhancing effect of epinephrine on IL-13 production (p < 0.00005 and p < 0.0001, respectively, Fig. 9C).

## Discussion

Inflammatory cytokines play an important role in atherogenesis. Acute phase response (APR) proteins have been demonstrated as risk factors for atherosclerotic heart disease [34]. Recent studies also suggest a prominent role for the APR in cerebrovascular disease and brain ischemia [35]. The APR culminates in the secretion of inflammatory cytokines such as IL-6, TNF-α", and IL-1 resulting in the synthesis of several proteins including C-reactive protein, fibrinogen, serum amyloid A protein, and ceruloplasmin [36,37]. These cytokines are intimately involved with the stress response [38]. These cytokines can also induce transcriptional regulation of complement genes that have been shown to play a role in cardiovascular disease [39]. Catecholamines are elaborated in stress responses which mediate vasoconstriction and elevate systemic vascular resistance and blood pressure. Catecholamines induce aggravation of aortic and coronary atherosclerosis in monkeys [20] and play a direct role in atherogenesis and cardiovascular disease [21]. Thus, it is important to understand the interaction between epinephrine and IL-1β with respect to atherogenic cytokine production.

In this study, IL-1β, an acute phase cytokine, activated mast cells to produce proatherogenic cytokines, IL-6, IL-8, and IL-13, in a dose-dependent manner (Fig 1, 2, and 3). These results confirm our previous report that IL-1β regulates mast cell function [40]. These results also show that epinephrine significantly up regulated the IL-1β induction of proatherogenic cytokines in mast cells giving new insight into neuronal regulation of the immune system. The gene expression of these proatherogenic cytokines was also increased in IL-1β-induced HMC-1 cells by addition of epinephrine, suggesting that the enhancing effect of proatherogenic cytokine production is a result of increased cytokine gene transcription (Fig. 4). These data provide a novel role for epinephrine in inflammation and atherogenesis.

IL-1β signaling probably synergizes with β_2_-adrenoreceptor-mediated signaling pathways in inducing proatherogenic cytokine production. Several reports have shown that the effect of catecholamines on immune function is due to β-adrenoceptors [41-45]. Flow cytometry data indicated that HMC-1 cells express both β_1 _and β_2 _adrenoceptors in small amounts (Fig. 5). The result showed that the enhancing effect of proatherogenic cytokine production by epinephrine is down regulated by β_1 _and β_2 _adrenoceptor antagonist, propranolol, but not by β_1 _specific adrenoceptor antagonist, atenolol, further suggesting the enhancing effect involves β_2 _adrenoceptors (Fig. 3). The down regulation by propranolol does not appear to be due to cytotoxicity of the antagonist since there is no difference in viabilities between the propranolol-treated and untreated cell cultures. It was interesting to see that propranolol caused a reduction of production of IL-13 to an amount that was much lower than that treated with IL-1β only (Fig. 3). It may be that epinephrine-induced enhancement of IL-13 production is more sensitive to the propranolol blocking.

Activated NF-κB has been demonstrated in atheromatous plaques and has been shown to play a role in atherogenesis [46]. To study the mechanism of the enhancing effect of epinephrine on proatherogenic cytokine production from IL-1β-induced mast cells, NF-κB and p38 MAPK activations were investigated. Control samples and epinephrine alone samples did not induce NF-κB activation. However, a marked increase in NF-κB activation was observed in samples stimulated with IL-1β and IL-1β plus epinephrine (Fig. 7). NF-κB activation was seen early at one hour and began to fade by two hours. NF-κB also was not increased by the addition of epinephrine to IL-1β and even seemed to decrease it at both time points suggesting that NF-κB is needed for cytokine induction but not for the enhancing effect. The presence of phosphorylated p38 MAPK was greatly increased in the epinephrine and IL-1β plus epinephrine samples but only minimally activated with IL-1β alone at a 30 minute incubation time point (Fig. 8). SB203580 blocked the IL-1β and IL-1β plus epinephrine effect on IL-6, IL-8, and IL-13 expression suggesting that p38 plays an important role in signaling from both IL-1β and epinephrine. The double stimulation of p38, early by IL-1β and later by epinephrine, may explain the enhancing effect on the production of IL-6, IL-8, and IL-13 in mast cells.

The enhancing effect of epinephrine on proatherogenic cytokine production was also down regulated by immunosupressants, such as Dex. Dex at the concentration used in this study did not affect the cell viability of the culture, suggesting the down regulation effect of the drugs is not due to toxic effect. Dex also slightly, but not significantly, decreased IL-1β-induced cytokine production in mast cells (Fig. 6). Taken all together, these results indicate that β_2_-adrenoceptor antagonists and glucocorticoids may have clinical potential in stress-mediated disease and atherogenesis.

**Figure 10:**
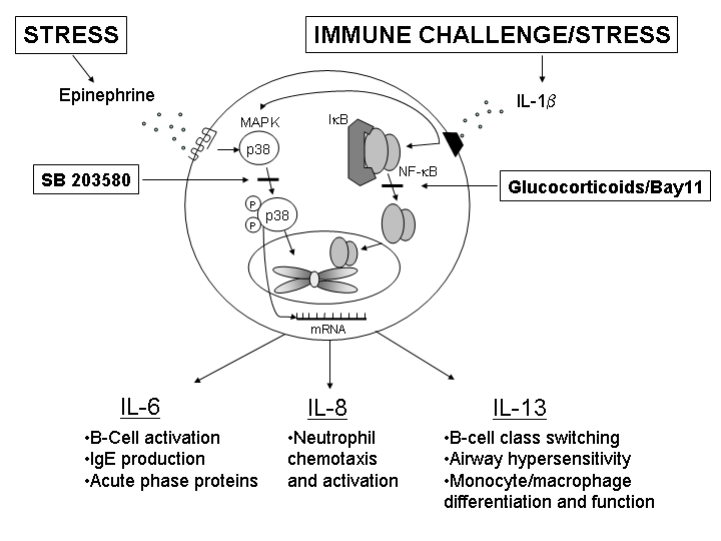
Schematic presentation showing the possible route of IL-6, IL-8, and IL-13 signaling. Endogenous IL-1β production may occur with immune challenge by cytokines, bacteria, and viruses, and any microtrauma in the body while epinephrine is released in states of stress or sympathetic nervous system activation. The pathways activated by these signals converge on IL-6, IL-8, and IL-13 genes to induce cytokine production that is greater than either signal alone. IL-1β activates the NF-κB pathway which leads to significant amounts of IL-6, IL-8, and IL-13 production. Epinephrine activates the p38 MAPK pathway which may activate other transcription factors or stabilize the IL-6, 8, and 13 mRNA. From our data it is evident that IL-1β and epinephrine do not combine to further activate NF-κB or the promotor activity of the IL-13 gene. The importance of IL-6, IL-8, and IL-13 are listed in the figure.

All the signaling pathways induced by IL-1β and epinephrine in mast cells are complex and beyond the scope of this manuscript. However, two important inflammatory pathways, NF-κB and p38 MAPK, have been shown. IL-1β release from immune challenge and epinephrine elevated from stress response can jointly stimulate mast cells to increase IL-6, -8, and -13 production above that which is seen with either stimulus alone. The exact mechanisms are unclear, but we have shown that IL-1β is a strong inducer of NF-κB while epinephrine is a strong inducer of p38 MAPK. Neither NF-κB nor p38 MAPK was activated further by IL-1β plus epinephrine compared to either stimulus alone nor was the promotor activity of IL-13 increased by the double stimulus as seen by luciferase activity of a IL-13 reporter gene construct. These data would suggest that IL-1β is activating IL-6, IL-8, and IL-13 by NF-κB while p38 MAPK activation is enhancing protein production by inducing other transcription factors, stabilizing the gene mRNA, or other forms of post-translational modification. These mechanisms are summarized in Fig. 10.

## Conclusions

In conclusion, stress related catecholamines, such as epinephrine, synergized with IL-1β in gene expression and production of proatherogenic cytokines, IL-6, IL-8, and IL-13 in mast cells. The enhancing effect of proatherogenic cytokine production by epinephrine on IL-1β-induced mast cells was down regulated by β-adrenoceptor antagonist, propranolol, and the immunosuppressant Dex. These data support a novel role for catecholamines in disorders such as inflammation and atherogenesis. These data also indicate that β-adrenoceptor antagonists and immunosuppressants may be used preventively and therapeutically for modulation of the catecholamine – proatherogenic cytokine axis in disease states.

## Methods

### Mast cell culture and the induction of cytokine production in HMC-1 cells

HMC-1 cell line, established from a patient with mast cell leukemia, were graciously provided by Dr. Butterfield (Mayo Clinic, Rochester, MN). These cells were maintained in RPMI 1640 media (GibcoBRL, Rockville, MD), supplemented with 5 × 10^-5 ^M 2-mercaptoethanol (Sigma Chemical Company, St. Louis, MO), 10 mM HEPES (GibcoBRL), Gentamycin 50 μg/ml, 5 μg/ml insulin transferrin, 2 mM L-glutamine, and 5% heat inactivated fetal bovine serum (Atlanta Biologicals, Atlanta, GA), at 37°C and in 5% CO_2 _mixture [33]. HMC-1 cells were cultured and maintained in 25 cm^2 ^flasks. To each well of a 6 well culture plate, two ml of HMC-1 mast cells at 0.75 × 10^6 ^cells/ml concentration were cultured with epinephrine at 1 × 10^-5 ^M concentration in the presence and absence of IL-1β (10 ng/ml) for 24 hrs. The cultures were carried out in triplicate. Supernatants were harvested for measuring IL-6, IL-8, and IL-13 by ELISA and cell viability and numbers of the culture were analyzed.

### ELISA for cytokine proteins

Cytokine ELISA was performed for the following cytokines: IL-6, IL-8, and IL-13. ELISA was carried out on cell-free culture supernatants using commercially available ELISA kits, according to manufacturers instructions as earlier described (R&D Systems, Minneapolis, MN; Immunotech, Westbrook, ME; Genzyme, Cambridge, MA). Results were analyzed on an ELISA plate reader (Dynatech MR 5000 with supporting software) [47,48].

### Measurement of cell viability of the cultures

At the end of incubation, the cells were subjected to the viability count by trypan blue (TB) dye exclusion technique. Two tenths ml of cell cultures were mixed with 0.05 ml of TB, applied to hemocytometer, and counted under a microscope. The cell viability is calculated by dividing the number of live cells (unstained cells) by the total number of all cells (TB-stained and unstained cells) and expressed as a percent.

### Analysis of cytokine gene expression by RT-PCR

HMC-1 were treated with the appropriate reagents and allowed to incubate at 37°C before being harvested for RNA. RNA was extracted from HMC-1 (3 × 10^6 ^cells) by the addition of 1 ml of RNAzol B (Tel-Test, Inc., Friendswood, Texas) [49]. After shaking for 1 minute the samples were centrifuged at 12,000 × g for 15 minutes at 4°C. The aqueous layer was washed twice with 0.8 ml phenol : chloroform (1:1, v/v), centrifuged at 12,000 × g for 15 minutes at 4°C, washed once with 0.8 ml of chloroform and centrifuged at 12,000 × g for 15 minutes at 4°C again. Isopropanol was added to the aqueous phase, and the preparation was frozen at -20°C overnight. The following day, the samples were centrifuged at 12,000 × g for 30 minutes at 4°C. The RNA pellet was washed with 1 ml 75% ethanol and allowed to air dry until all moisture was gone. The pellet was resuspended in DEPC water and quantitated by optical density readings at 260 nm. cDNA was synthesized with murine leukemia virus reverse transcriptase (2.5 U/μl), 10 × PCR buffer (500 mM KCl, 100 mM Tris-HCl, pH 8.3), 1 mM each of the nucleotides dATP, dCTP, dGTP and dTTP; RNase inhibitor (1 U/μl), MgCl_2 _(5 mM), and oligo(dT)_16 _(2.5 μM) as a primer. The samples were incubated at 42°C for 20 minutes, 99°C for 20 minutes, and 5°C for 5 minutes in a DNA thermocycler (Perkin-Elmer Corp., Norwalk, CT) for reverse transcription. PCR of cDNA was done with MgCl_2 _(1.8 mM), each of the dNTPs (0.2 mM), AmpliTaq polymerase (1 U/50 μl), and paired cytokine-specific primers (0.2 nM of each primer) to a total volume of 50 μl. Cycles consisted of 1 cycle of 95°C for 2 min, 35 cycles of 95°C for 45 sec, 60°C for 45 sec, and 72°C for 1 min 30 sec, and lastly, 1 cycle of 72°C for 10 min. Ten microliters of the sample were electrophoresed on a 2% agarose gel and stained with ethidium bromide for viewing. Primer sequences used are as follows: HPRT: 5' CGA GAT GTG ATG AAG GAG ATG G 3' and 5' GGA TTA TAC TGC CTG ACC AAG G 3'; IL-6: 5' ATG AAC TCC TTC TCC ACA AGC GC 3' and 5' GAA GAG CCC TCA GGC TGG ACT G 3'; IL-8: 5' ATG ACT TCC AAG CTG GCC GTG GCT 3' and 5' TCT CAG CCC TCT TCA AAA ACT TCT C 3'; and IL-13: 5' GGA AGC TTC TCC TCA ATC CTC TCC TGT T 3' and 5' GCG GAT TCG TTG AAC CGT CCC TCG CGA AA 3'. Densitometry was done by normalizing target genes to house keepers using Un-Scan-It Version 5.1 software (Orem, UT). The PCR experiment was repeated twice.

### NF-κB assay in HMC-1

HMC-1 were stimulated with PMA, IL-1β and/or epinephrine and then harvested for EMSA analysis [49,50]. Cells were washed with PBS and mixed with one hundred microliters of hypotonic buffer which contains: 10 mM HEPES pH 7.9, 10 mM KCl, 0.1 mM EDTA, 0.1 mM EGTA, 1 mM dithiothreitol (DTT), 0.5 mM phenylmethylsulfonyl fluoride (PMSF), 1 μM aprotinin, 1 μM pepstatin, 14 μM leupeptin, 50 mM NaF, 30 mM β-glycerophosphate, 1 mM Na_3_VO_4_, and 20 mM p-nitrophenyl phosphate. Cells were incubated over ice for 30 minutes and then vortexed after the addition of 6.25 μl of 10% of Nonidet P-40. After 2 minutes of centrifugation at 30,000 × g, supernatants were kept at -80°C while the pellets were collected and vortexed every 20 minutes for 3 hours in 60 ml of a hypertonic salt solution: 20 mM HEPES pH 7.9, 0.4 M NaCl, 1 mM EDTA, 1 mM EGTA, 12 mM DTT, 1 mM PMSF, 1 μM aprotinin, 1 μM pepstatin, 14 μM leupeptin, 50 mM NaF, 30 mM β-glycerophosphate, 1 mM Na_3_VO_4_, and 20 mM p-nitrophenyl phosphate. Nuclear translocation of NF-κB was analyzed by the Electrophoretic Mobility Shift Assay (EMSA) using the nuclear fraction. Seven micrograms of nuclear protein were added to 2 ml of binding buffer (Promega, Madison, WI), and 35 fmol of double stranded NF-κB consensus oligonucleotide (5' AGT TGA GGG GAC TTT CCC AGG C 3') (Promega, Madison, WI) end labeled with γ-P^32 ^ATP (Amersham Biosciences, Piscataway, NJ). The samples were incubated at room temperature for 20 minutes and run on a 5% nondenaturing polyacrylamide gel for 2 hours. The gel was then dried on a Gel-Drier (Bio-Rad Laboratories, Hercules CA) and exposed to Kodak X-ray film at -80°C.

### Detection of p38 MAPK by Western blot

Cells were treated and lysed in lysis buffer (50 mM Tris HCL, 150 mM NaCl, 1 mM EDTA, 1% Triton × 100, and 0.1% SDS) to be analyzed for p38 MAPK activation by Western blot [29]. Briefly, 50 μg of sample protein was diluted 1:2 with Laemmli buffer (Bio-Rad laboratories, Hercules, CA) and boiled for 10 minutes in a sand bath. The resultant sample was then run in a Bio-Rad Modular Mini Electrophoresis System (Hercules, CA) on a 10% polyacrylamide gel for 1 hour and then transferred to a 0.2 μm nitrocellulose membrane (Bio-Rad laboratories, Hercules, CA) for 1 hour. The blot was then incubated in blocking buffer (1% BSA and 0.1% Tween in PBS) for 1 hour at room temperature with gentle agitation. Rabbit anti-human Phospho-p38 MAPK (Thr180/Tyr182) polyclonal antibody (Calbiochem, San Diego, CA) was diluted 1:1000 in blocking buffer and incubated on the blot overnight at 4°C with gentle agitation. After the primary antibody was removed the blot was washed three times for 10 minutes each with agitation in the wash buffer (0.1% Tween in PBS). The blot was then incubated in horse radish peroxidase conjugated mouse anti-rabbit Ig's antibody (human adsorbed, Santa Cruz Biotechnology, Santa Cruz, CA) diluted 1:5000 in blocking buffer. The blot remained in the secondary antibody for 1 hour at room temperature. The blot was then washed again and covered with Super Signal West Pico Chemiluminescent Substrate (Pierce, Rockford, IL) for 5 minutes. The blot was then exposed to acetate transparency film (Kodak, Rochester, NY) and developed. The same protocol was repeated for total p38 MAPK analysis.

### Analysis of β-adrenoceptor by flow cytometry

Resting HMC-1 were centrifuged, washed in PBS at room temperature, and resuspended in 100 μl of PBS. The cells were incubated for 20 minutes with rabbit polyclonal anti β_1 _or β_2 _adrenergic receptor antibodies (Santa Cruz, Santa Cruz, CA) using normal rabbit serum as a control. The samples were washed with PBS with 0.01% sodium azide and resuspended in 100 ml PBS. FITC labeled goat anti-rabbit Ig's antibody was added to the samples and allowed to bind for 20 minutes. The samples were once again washed with PBS with 0.01% sodium azide and resuspended in 100 μl of PBS. In addition, HMC-1 were pretreated with normal rabbit serum and incubated with FITC labeled goat anti-rabbit Ig's antibody as a control for nonspecific binding [51]. Cell suspensions were then gated and analyzed based on fluorescence using a Becton Dickinson FACSCalibur 4-color flow cytometer (San Diego, CA) and histograms generated on WinMDI 2.8 software (kindly provided by Joseph Trotter over the internet).

### IL-13 promotor analysis

HMC-1 were treated with IL-1β (10 ng/ml), epinephrine (10^-5 ^M), and IL-1β plus epinephrine to investigate IL-13 promotor activity. Untreated cells were used as a control. Transient transfection assays were performed using a reporter gene construct containing the minimal promoter sequence of IL-13. The promoter sequence (-233 to + 50, relative to the transcription initiation site) of the IL-13 gene was fused to the luciferase coding sequence. Plasmid DNA was obtained with double-cesium chloride purification (BioServe Biotechnologies, Laurel, MD), while SuperFect reagent (Qiagen) was used for transient transfections of HMC-1 cells. Two micrograms of plasmid DNA and 8 μl SuperFect reagent were used for transfection of 1 × 10^6 ^HMC-1 cells. Luciferase expression was monitored by chemiluminescence of cell lysates 24 hrs after transfections using the Enhanced Luciferase Assay Kit (Analytical Luminescence Laboratory, Ann Arbor, MI).

### Statistical analysis of the data

All experiments were done in triplicate. The data were analyzed by Student's two-tailed *t*-test using Statistica software (StatSoft, Inc., Tulsa, OK). All data were reported as means ± SE. A *p*-value of less than 0.05 was considered significant.

## List of abbreviations used

HMC-1, human mast cell – 1

Epi, epinephrine

Pro, propranolol

Ate, atenolol

Dex, dexamethasone

MAPK, mitogen-activated protein kinase

## Author's contributions

DSC designed experiments, oversaw research, and wrote paper. SMF designed and conducted experiments and wrote paper. SP helped with experiments. KC conducted experiments. EK conducted experiments. SAL conducted experiments. SKH conducted experiments. GK oversaw research.
